# Supplementary material for: Folate Receptor Targeted Photodynamic Therapy: A Novel Way to Stimulate Anti-Tumor Immune Response in Intraperitoneal Ovarian Cancer
Source: Int J Mol Sci. 2023 Jul 10;24(14):11288. doi: 10.3390/ijms241411288 (PMC10378870; doi:10.3390/ijms241411288)
Supplement: Supplementary file 1 [file ijms-24-11288-s001.zip › ijms-2305636-supplementary.pdf]

<u>**Supplementary materials :**</u>

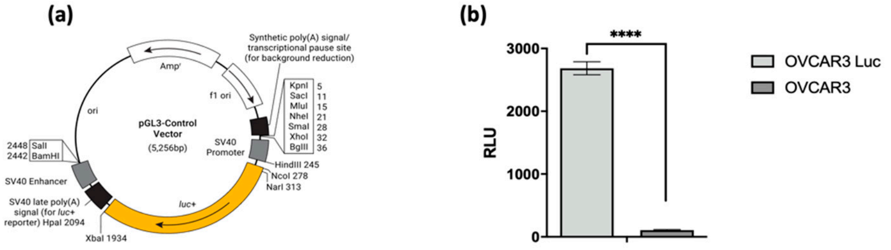

Figure S1. Validation of Luciferase expression: (**a**) pGEM®-luc vector GenBank Accession Number X65316: Luc-plasmide transfected into OVCAR3 cell line; (**b**) luminescence emitted by OVCAR3-Luc cell-line compared to classical OVCAR3 cell-line; results are presented as means of three independent experiments, expressed in Relative Light Unit (RLU). Student T statistical test was performed, p-value is two – sided, with $p \leq 0.0001$ (****) being considered statistically highly significant. $n = 3$

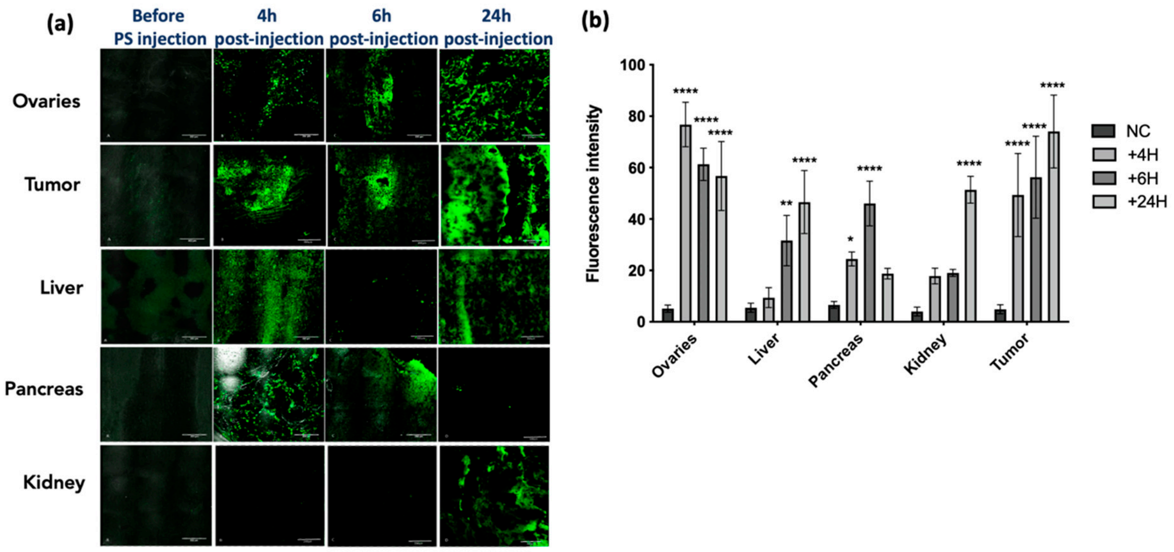

Figure S2: Evaluation of the uptake of the Pyro-PEG-FA, **(a)** PS uptake in an *in vivo* humanized SCID mice model of intraperitoneal ovarian cancer within ovaries, tumour, liver, pancreas and kidney (scale bar = 200 µm), **(b)** Semi Fluorescence quantification of the signal by ImageJ software (NC: Negative control). Results are expressed in fluorescence intensity 4h, 6h and 24h after PS injection and presented as means of three independent experiments. 2-way ANOVA statistical test was performed, all quoted *p*-values are two-sided, with $p \leq 0.05$ (*), $p \leq 0.01$ (**) and $p \leq 0.0001$ (****) being considered statistically significant for the first and highly significant for the others. $n = 3$.

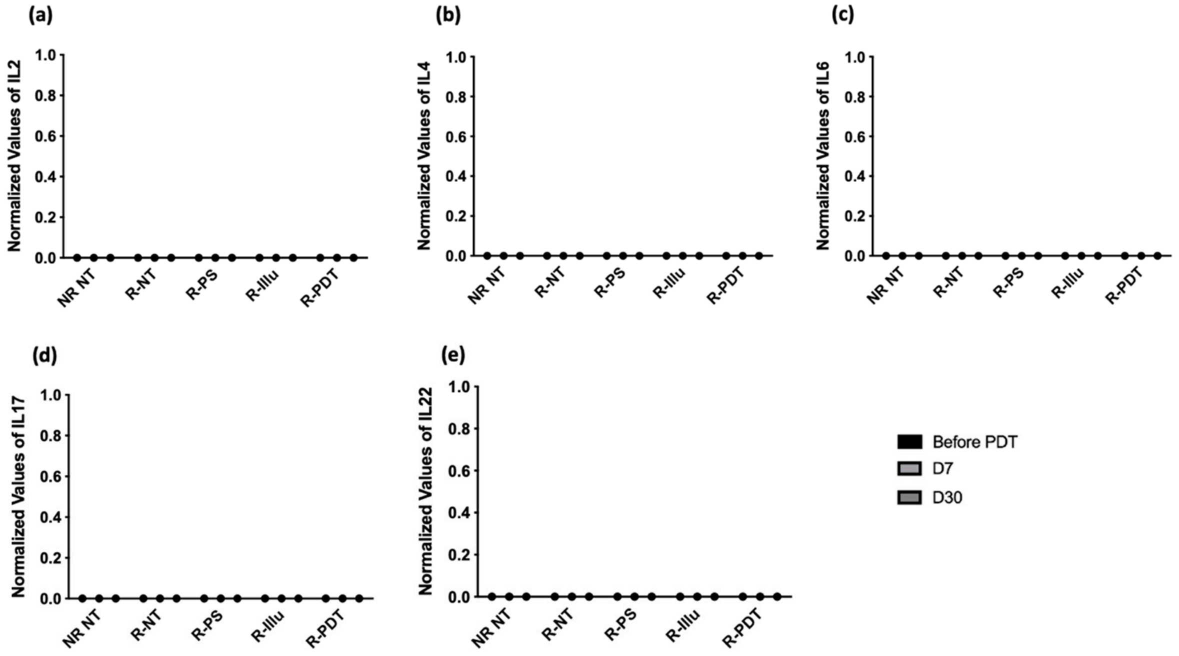

Figure S3. Evaluation of the cytokine release upon PDT in PBMC-reconstituted humanized SCID mice model of peritoneal ovarian cancer: Blood from not reconstituted (NR NT) or reconstituted mice subjected to illumination (R-PDT, R-Illu) or not (R-PS, R-NT) was examined for cytokine release 7 days (D7) and 30 days (D30) after PDT: (**a**) Interleukine 2 (IL-2); (**b**) Interleukine 4 (IL-4); (**c**) Interleukin 6 (IL-6); (**d**) Interleukine 17 (IL-17*)*; (**e**): Interleukine 22 (IL-22) with NRNT : non – reconstituted non treated, R-NT : reconstituted non treated, R-PS : Reconstituted subjected to Photosensitizer only, R-illu : Reconstituted subjected to Illumination only, R-PDT : Reconstituted subjected to Illumination in the presence of Photosensitizer.
